# Comprehensive Management of Daily Living Activities, behavioral and Psychological Symptoms, and Cognitive Function in Patients with Alzheimer's Disease: A Chinese Consensus on the Comprehensive Management of Alzheimer's Disease

**DOI:** 10.1007/s12264-021-00701-z

**Published:** 2021-05-29

**Authors:** Jianjun Jia, Jun Xu, Jun Liu, Yongjun Wang, Yanjiang Wang, Yunpeng Cao, Qihao Guo, Qiuming Qu, Cuibai Wei, Wenshi Wei, Junjian Zhang, Enyan Yu

**Affiliations:** 1 Department of Neurology, The Second Medical Center, People’s Liberation Army General Hospital, Beijing, 100853 China; 2 Department of Neurology, Beijing Tiantan Hospital, Capital Medical University, Beijing, 100050 China; 3 Department of Neurology, Sun Yat-sen Memorial Hospital, Sun Yat-sen University, Guangzhou, 510120 China; 4 Cognitive Impairment Department, Shenzhen Kangning Hospital, Shenzhen, 518118 China; 5 Department of Neurology, Daping Hospital, Army Medical University, Chongqing, 400042 China; 6 Department of Neurology, The First Hospital of China Medical University, Shenyang, 210112 China; 7 Department of Gerontology, Shanghai Jiaotong University Affiliated Sixth People’s Hospital, Shanghai, 200233 China; 8 Department of Neurology, The First Affiliated Hospital of Xi’an Jiaotong University, Xi’an, 710061 China; 9 Department of Neurology, Xuanwu Hospital Capital Medical University, Beijing, 100053 China; 10 Department of Neurology, Huadong Hospital Affiliated to Fudan University, Shanghai, 200040 China; 11 Department of Neurology, Zhongnan Hospital of Wuhan University, Wuhan, 430071 China; 12 Department of Psychology, Chinese Academy of Sciences Cancer Hospital of the University of the Chinese Academy of Sciences, Hangzhou, 310022 China

**Keywords:** Alzheimer's disease, Comprehensive management, Activities of daily living, Behavioral and psychological symptoms, Cognitive function

## Abstract

Alzheimer's disease (AD) is the most common cognitive disorder in the elderly. Its main clinical manifestations are cognitive decline (C), behavioral and psychological symptoms (B), and a decline in the activities of daily living (A), also known as ABC symptoms. Early identification and evaluation of ABC symptoms are helpful for establishing the accurate diagnosis, comprehensive treatment, and prognosis of AD. To guide Chinese clinical practice for optimization of the comprehensive management of AD, in 2018, The Academy of Cognitive Disorder of China gathered 22 neurologists and gerontologists in China to build a consensus on the comprehensive management of AD. Based on a review of the evidence, the consensus summarizes the pathogenesis, pathological changes, clinical manifestations, evaluation, diagnosis, drug and non-drug treatment, and patient care for AD. Focus group discussion was used to establish a flowchart of comprehensive ABC management for AD patients. The new consensus provides a feasible AD management process for clinicians.

Alzheimer’s disease (AD) is the most common cognitive disorder in the elderly. Epidemiological surveys show that the prevalence of AD is 3.21% among people ≥65 years in China [1]. It is estimated that the total costs of AD in China will be $248.71 billion in 2020, $507.49 billion in 2030, $1.00 trillion in 2040, and $1.89 trillion in 2050 [2]. The number of patients who died of AD in the last 10 years increased by 57.8% in China, making AD the sixth leading cause of death in 2016 [3]. The major clinical manifestations of patients with AD include cognitive impairment (C), psychiatric and behavioral symptoms (B), and reduced activities of daily living (A), summarized as the ABC symptoms. Various complications occur in patients in the moderate or severe stages of AD, requiring long-term comprehensive management and imposing heavy economic burdens on the families and society [2]. Conducting active and integrated AD management is hence necessary. Therefore, the Academy of Cognitive Disorder of China initiated this expert consensus on the comprehensive ABC management of patients with AD, aiming to optimize the procedures of AD diagnosis and enhance the comprehensive management for clinicians.

## Estimated Demographics of AD Patient in China

**Fig. 1:**
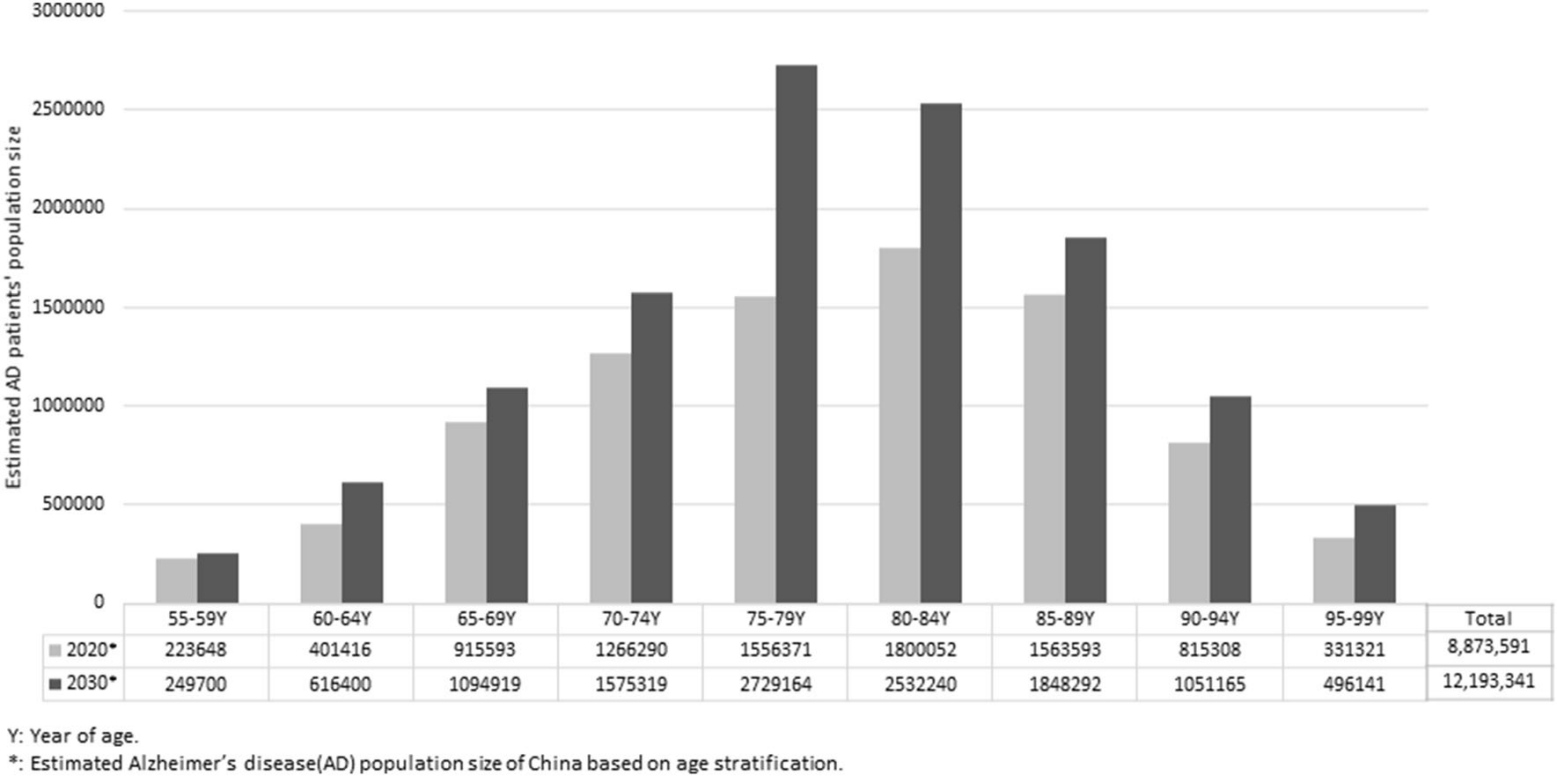
Estimated Alzheimer’s disease population sizes in China based on age stratification.

The etiologies of AD are still unclear. However, AD can be diagnosed based on the symptoms and other tests illustrated in the diagnostic criteria. The reported prevalence data provide a cost-effective way to estimate the demographics of AD patients for estimating the disease burden. The general estimated population size of AD has been reported in other studies, while estimation of the sizes of AD populations based on age stratification is needed for further precise disease management. The percentage of people with Alzheimer’s dementia increases with age. People younger than 55 can also develop AD, but it is much less common and more related to genetic factors. We focused on the demographics of AD patients aged from 55 to 99. Based on the data from the population censuses of 2010 and 2000, which reported the prevalence of AD in different age groups, we estimated the AD population size in different age groups for 2020 and 2030. The 10-year survival rate referred to the 10-year survival rate from 2000 to 2010 and the prevalence of AD for each age group was 0.23% (55–59 years), 0.55% (60–64), 1.27% (65–69), 2.73% (70–74), 5.52% (75–79), 10.44% (80–84), 18.54% (85–89), 30.86% (90–94), and 48.19% (95–99) [4]. The number of AD patients in the next decade will dramatically increase (Fig. 1).

## Pathogeneses and Pathological Changes in AD

The etiologies of AD are still unclear. However, studies have demonstrated that its development is affected by various factors, including aging, heredity, lifestyle, and environment [5–8].

### Beta-amyloid and Tau

The deposition of beta-amyloid (Aβ) is believed to be the core of AD pathogenesis [9]. A new study suggests that Aβ-dependent neuronal hyperactivation is initiated by the suppression of glutamate reuptake before plaque formation [10]. Hyperphosphorylated tau is induced by various pathological factors, and, in turn, leads to abnormal Aβ accumulation.

### Neuroinflammation

Neuroinflammation is associated with the neurodegeneration of AD and it may precede neurodegeneration. The triggers of inflammation include Aβ, neurodegeneration, and infection of the brain and elsewhere in the body that activates the immune system in the brain [11]. Studies with controversial conclusions have found that human herpesvirus (HHV) types 6A, 6B, and 7 are linked with AD [12–14]. One observational study with a 12-year follow-up showed that patients >50 years old with newly-diagnosed HSV-1 or HSV-2 infection have a higher risk of AD than patients with no HSV infection, and antiviral treatment can reduce the risk of dementia [15]. So, while neuroinflammation treatment might be a promising way to conquer AD, more studies are needed.

### Genetics

Several genes have been found that increase the risk of AD, while the apolipoprotein-e4 (APOE-e4) gene has the strongest impact on the risk of late-onset AD. Those who inherit one copy of *APOE-e4* have about 3 times the risk of developing AD, while those who inherit two copies have an 8-to-12-fold risk [16–18]. Autosomal dominant familial forms of AD are due to mutations in one of three genes: amyloid precursor protein (APP), presenilin (PSEN) 1, and PSEN2 on chromosomes 21, 14, and 1, respectively. Genetic factors most likely determine the rate of disease progression (e.g., the brain-derived neurotrophic factor polymorphism) [19, 20]. In Han Chinese, a study showed that APOE-e4, the RS2305421 GG genotype, and the RS10498633 GT genotype are associated with the Aβ plaque score, Braak neurofibrillary tangle stage, and CERAD (Consortium to Establish a Registry for Alzheimer's disease) neuritic plaque score; these results have advanced our understanding of the pathogenesis of AD [21].

## Clinical Manifestations of AD Patients

### ABC Symptoms of AD

The clinical manifestations of ABC symptoms are summarized in Table 1 [22–24].

**Table 1:** Symptoms of reduced activities of daily living (A), psychiatric and behavioral symptoms (B), and cognitive impairment (C) of patients with Alzheimer’s disease.

Symptoms	Type	Detailed contents, items, or manifestations
Reduced activities of daily living	Basic activities of daily living	Defecation, feeding, dressing/undressing, grooming, walking, and bathing
	Intellect activities of daily living	Using telephone, shopping, cooking, doing housework, washing clothes, taking a bus independently, taking medicine, and financial independence
Psychiatric and behavioral symptoms	Apathy/Indifference	Decreased concern about daily activities and self-management; evidently reduced socialization, facial expression, verbal communication, and emotional responses; and absence of motivation
	Agitation/Offensive	Offensive behaviors, including scratching, biting, and kicking; non-offensive body behaviors, including screaming, resisting, defending, and self-protecting movements; non-offensive language
	Depression/Dysthymia	Negative emotions, including low mood, pessimism, sense of helplessness, and sense of hopelessness
	Anxiety	Repeatedly asking questions or fear of being alone; some patients also fear crowds, traveling, darkness, or bathing
	Irritability/Emotional lability	Irritable, quick mood changes, and extremely impatient
	Hyperthymia/Euphoria	Over-happy, feeling too good, feeling funny and laughing at things that are not interesting to others, or showing scenario-inconsistent happiness
	Appetite and eating disorders	Weight gain or loss, and changes in the flavor of enjoyed food
	Sleep/Nocturnal behaviors	Circadian rhythm disorder, increase in waking after sleep onset at night, and rapid eye movement sleep behavior disorder
	Hallucination	Including visual and auditory hallucinations, with visual hallucination more common; the most common visual hallucination involves seeing people who do not exist in the home or seeing deceased relatives
	Delusion	Five typical delusions: items being stolen, living in another’s house, suspicious of spouse (or caregiver), being abandoned, and unfaithful spouse
	Abnormal motor behaviors	Wandering aimlessly or following the caregiver all day, and requiring to go out at night
	Disinhibition	Abrupt behaviors: naturally engaging with strangers, not considering others’ feelings, and behaviors violating social morality
Cognitive impairment	Learning and remembering	Immediate memory and recent memory (free recall, cued recall, and recognition)
	Language	Expressive language (naming, fluency, grammar, and syntax) and receptive language
	Executive capability	Planning, decision-making, working memory, capability of feedback and correction, habit inhibition, and flexibility
	Composite attention	Sustained attention, divided attention, selective attention, and processing speed
	Visuoperceptual function	Structure and visuoperceptual function
	Social cognition	Emotion recognition, psychological inference, and behavior regulation

Usually, the first symptom is memory impairment, which can be manifested as forgetting and loss of the capacity to learn new information, such as repeatedly asking the same question, misplacing stuff, or even forgetting important events [25]. Language problems include not only difficulties in naming and/or finding words, grammatical and/or syntax errors, and impaired linguistic coherence and logicality but also difficulty with comprehension, writing errors, and communication disorders. Language problems consequently prevent patients from communicating with others. Executive dysfunction can appear in the early stage; this includes impairments of abilities of inference and handling complex tasks; reduction in judgment, financial management, and decision-making; and decreased socialization and working competence. Mild composite attention dysfunction is manifested as requiring a longer time for routine tasks than previously, as well as making mistakes in work. Difficulties in mental arithmetic or remembering and reciting new information may appear when composite attention dysfunction worsens. Visuospatial dysfunction is manifested as the absence of perception of the surrounding environment (including time, space, and people) and self-status (including name, age, and occupation), getting lost in familiar places, and being unable to recognize faces and regular objects. Social cognition impairment is manifested as personality changes and inconsiderate and unacceptable daily behaviors.

Subjective cognitive decline refers to patients believing that their memory and cognitive functions have declined compared with their previously normal status, while they are within the normal ranges measured using objective neuropsychological tests. These patients may be in the preclinical stage with AD-like physiological changes [26].

The psychiatric and behavioral symptoms can appear in any stage of AD, although the incidence of agitation, anxiety, delusion, and abnormal behaviors is higher in patients with moderate than in those with mild AD. The mild behavioral impairments in patients with preclinical AD include emotional instability, impulse control disorder, social discomfort, and abnormal beliefs and ideas. People with mild behavioral change show increased risk of developing AD.

Activities of daily living (ADL) include basic (BADL) and intellectual activities (IADL). Feeding and bathing are significant risk factors for an increases caregiver burden [27].

## Associations Among the ABC Symptoms

Cognitive impairment is associated with IADL reduction in the early stage of AD, [28] while the worsening of cognitive function and BADL reduction are closely connected in the late stage [29]. The executive capability and personality changes may be independent risk factors in reducing IADL [30]. Therefore, ADL is important for the identification and diagnosis of cognitive impairment, and ADL assessment helps predict the risk of cognitive impairment development [31]. Also, the reduction in BADL and IADL is associated with visuospatial dysfunction [32]. Early ADL assessment should be conducted in patients with AD.

Studies have shown that the Mini-Mental Status Examination (MMSE) score is associated with the Neuropsychiatric Inventory (NPI) score, suggesting that cognitive impairment can be used to predict the behavioral and psychological symptoms of dementia (BPSD) [33]. The severity of BPSD is associated with ADL reduction; however, patients with stable BPSD do not suffer from a dramatic cognitive decline regardless of the disease stage [34].

The ABC symptoms are closely associated and interact with each other; [31–33] even in very mild AD, executive function, depression, and apathy are associated with IADL decline among female patients [35]. It is rational for clinicians to identify and assess ABC symptoms comprehensively. Early identification and assessment of ABC symptoms help to accurately diagnose and comprehensively treat AD as well as predicting the prognoses of patients.

### Other Phenotypes of AD

Other phenotypes of AD include posterior cortical atrophy (PCA), logopenic progressive aphasia (LPA), frontal variant AD, and trisomy-21 syndrome. Although their clinical manifestations vary, the pathological features suggest that they are all AD. Visuospatial and visuoperceptual dysfunctions appear in patients with PCA without evident visual causes. The capability of word expression decreases in patients with LPA, while the abilities of grammar, pronunciation, and motor speech are preserved. The speech rate of patients with LPA is low; patients frequently pause due to looking for words. The wording is generally correct despite the simple syntactic structures. Patient with LPA usually have difficulties in comprehending and reciting complex sentences. Their naming function might be damaged. Frontal symptoms, such as behavioral abnormalities, including indifference and behavioral disinhibition, occur in the early stage of frontal variant AD. Besides, psychiatric symptoms, such as delusions, also occur. The executive functions of the patients are evidently impaired, followed by temporoparietal symptoms, such as memory deterioration, reduction in calculation ability, and spatial dysfunction. Notable mental retardation has also been found in patients with the trisomy-21 syndrome, which is accompanied by physical retardation, and specific facial features.

## Comprehensive Assessment of ABC Symptoms

Currently, the assessment of ABC symptoms mainly depends on neuropsychological scales. Therefore, selecting the appropriate scales is critical for the early intervention and comprehensive management of AD. The appropriate scales for early AD screening should have the following characteristics: (1) cover the three aspects of ABC symptoms as much as possible; (2) have high sensitivity, specificity, and repeatability; and (3) be convenient and time-saving, with a time for completion <10 min if possible, which allows informants or the elderly to finish the scales independently. The scales suitable for community screening mainly include the Informant Questionnaire on Dementia (such as AD8), Informant Questionnaire on Cognitive Decline in the Elderly, and Measurement of Everyday Cognition.

The commonly-used cognitive function screening scales mainly include MMSE, Montreal Cognitive Assessment (MoCA), Montreal Cognitive Assessment-Basic (MoCA-B), and the Clock Drawing Task, while the diagnostic scales mainly comprise various cognitive aspects, including memory, language, attention, visuospatial, and executive functions. The Alzheimer’s Disease Assessment Scale is mainly used for the assessment of treatment efficacy, while the NPI is generally used for the assessment of BPSD. Mild behavioral abnormalities are identified using the Chinese or English version of the Mild Behavioral Impairment Checklist (the sensitivity and specificity are 86.96% and 86.00%, respectively) [36]. Activity of daily living is mainly assessed using ADL and Pfeffer Functional Activities Questionnaire. Comprehensive assessment scales mainly include the Clinical Dementia Rating and ABC-Dementia Scale [24, 37].

Mild cognitive impairment (MCI) is the transitive state between normal cognition and dementia. MCI refers to the state of progressive impairment of memory and other cognitive functions, while the ADL is generally not affected, and the patients do not meet the diagnostic criteria of dementia [38]. Early identification and diagnosis may favor early intervention in MCI to decelerate its progression to dementia, and help the families to establish the coping strategies early [39]. The Auditory Verbal Learning Test, Animal Fluency Test, Boston Naming Test, and Trail Making Test are commonly used for the assessment of MCI.

Recommendations: (1) comprehensive, systemic assessment should be conducted to assess cognitive functions, ADL, and BPSD; (2) the ADL scale is recommended for the assessment of ADL; the MMSE scale is recommended for the assessment of global cognition level; and the NPI scale is recommended for the assessment of BPSD (Table 2); (3) other suitable scales should be selected according to the conditions; and (4) a database of the ABC symptoms of Chinese patients with AD should be constructed as soon as possible, and the validity and reliability of the related scales for the diagnosis in Chinese patients should be assessed.

**Table 2:** Reference values of recommended tools for assessment of clinical manifestations.

Tools	Reference values
ADL	Full score of 64 points, <16 points is completely normal. Score of 2-4 points for single items indicates decrease of function. Score of ≥3 points on 2 or more items or a total score ≥22 points indicate obvious dysfunction.
NPI	No reference values for NPI. It is used to assess 12 behavioral disturbances. Both the frequency and severity of each behavior are determined.
MMSE	Full score of 30 points. For illiterate group ≤17 points, primary school group ≤20 points, high school or above group ≤24 points indicates cognitive impairment.

ADL, Activities of daily living; NPI, Neuropsychiatric Inventory; MMSE, Mini-Mental Status Examination.

## Diagnosis of AD

### Diagnostic Criteria

The accurate diagnosis of AD depends on the collection of complete medical history and physical examination data, as well as the comprehensive assessment of ABC symptoms. Imaging examinations, including magnetic resonance imaging (MRI) structural imaging, cerebral positron emission computed tomography (PET), single-photon emission computed tomography (SPECT), measurement of biomarkers in blood and cerebrospinal fluid, and other auxiliary examinations, help in improving the diagnostic accuracy and further determining the subtypes.

Since the issuing of the first diagnostic criteria by the US National Institute of Neurological Disorders and Stroke-Alzheimer Disease and Related Disorders in 1984, seven editions of diagnostic criteria have been successively released. In recent years, the diagnostic criteria for biomarkers have attracted universal attention. However, several practical challenges are faced, including difficulties in specimen collection and high prices, in clinical practice. The National Institute on Aging and Alzheimer’s Association released updated diagnostic recommendations for the preclinical, mild cognitive impairment, and dementia stages of Alzheimer’s disease in 2018 to help the performance of scientific research. This research framework focuses on the diagnosis of AD with biomarkers in living persons for clinical research [40].

### Biomarkers for Diagnosis and Discrimination

Cerebrospinal fluid and PET biomarkers of Aβ and tau are highly accurate for detecting AD pathology, but the high cost, invasiveness, and low availability of these tools restrict their widespread use as clinical diagnostic tools. More and more studies have focused on blood-based biomarkers for clinical use and for facilitating clinical trial recruitment and monitoring. These studies’ outcomes have provided many hints on facilitating the earlier and more accurate diagnosis of AD (Table 3).

**Table 3:** Blood-based biomarkers for AD diagnosis and discrimination.

Study	Cohorts	Biomarkers	Outcome
Karikari [41]	TRIAD and BioFINDER-2	Plasma p-tau181, Serum p-tau181	Diagnosis of AD (serum AUC = 95.91%, plasma AUC = 90.06%)
	TRIAD	Plasma p-tau181	Distinguishes AD from Aβ-negative young adults (AUC = 99.4%)
	BioFINDER-2	Plasma p-tau181	Distinguishes AD from vascular dementia (AUC = 92.13%)
	TRIAD and BioFINDER-2	Plasma p-tau181	Distinguishes AD from other neurodegenerative disorders (AUC = 82.76%–100%)
	BioFINDER-2	Plasma p-tau181	Distinguishes AD from PSP or CBS (AUC = 88·47%)
	TRIAD and BioFINDER-2	Plasma p-tau181	Distinguishes AD from CU older adults (AUC = 90.21%–98.24%)
	BioFINDER-2	Plasma p-tau181	Distinguishes AD from PD or MSA (AUC = 81·90%)
Jia [42]	28AD/25aMCI/29 healthy controls	Plasma Aβ42	Distinguishes AD from healthy older adults (AUC = 93%)
		Plasma Aβ43	Distinguishes AD from aMCI (AUC = 83%)
		Plasma Aβ44	Distinguishes aMCI from healthy older adults (AUC = 74%)
		Plasma T-tau	Distinguishes AD from healthy older adults (AUC = 89%)
		Plasma T-tau	Distinguishes AD from aMCI (AUC = 72%)
		Plasma T-tau	Distinguishes aMCI from healthy older adults (AUC = 79%)
		Plasma p-T181-tau	Distinguishes AD from healthy older adults (AUC = 88%)
		Plasma p-T181-tau	Distinguishes AD from aMCI (AUC = 76%)
		Plasma p-T181-tau	Distinguishes aMCI from healthy older adults (AUC = 73%)
Fotuhi [43]	45 AD/36 healthy controls	Plasma BACE1-AS	Distinguishes full-AD from healthy older adults (AUC = 98%)
		Plasma BACE1-AS	Distinguishes pre-AD from healthy older adults (AUC = 89%)
		Plasma BACE1-AS	Distinguishes full-AD/pre-AD from healthy older adults (AUC = 99%)
Nakamura [44]	JNCGG:121 and AIBL:252	Plasma APP699-711/Aβ1-42 and Aβ1-40/Aβ1-42	Distinguishes brain Aβ positive or negative (AUC = 96.7% for JNCGG, AUC = 94.1% for AIBL)

AUC, area under the receiver operating characteristic curve; TRIAD, 27 young adults, 113 cognitively unimpaired older adults, 45 MCI, 33 AD, 8 FTD; BioFINDER-2, 337 cognitively unimpaired older adults, 191 MCI, 126 AD, 18 Behavioural variant FTD or PPA, 36 PD or MSA, 12 Vascular dementia, 21 PSP or CBS; CU, cognitively unimpaired; MCI, mild cognitive impairment. FTD, frontotemporal dementia; PPA, primary progressive aphasia; PD, Parkinson’s disease; MSA, multiple systems atrophy; PSP, progressive supranuclear palsy; CBS, corticobasal syndrome; BACE1-AS, beta-amyloid cleaving enzyme 1 antisense; aMCI, amnestic mild cognitive impairment; pre-AD (MMSE ≥20), full-AD (MMSE <20); JNCGG, Japanese National Centre for Geriatrics and Gerontology, 62CU/30MCI/29AD; AIBL, Australian Imaging, Biomarker and Lifestyle Study of Ageing, 156CU/67MCI/29AD; PIB-PET, 11C-labelled Pittsburgh compound-B positron-emission tomography.

Recommendations: (1) a review of disease history and comprehensive assessment of ABC symptoms should be the basis for AD diagnosis; (2) imaging examinations, including MRI structural imaging, cerebral PET, and SPECT, can be auxiliary examinations for diagnosing AD; and (3) biomarker examinations, including screening for the apolipoprotein E ε4 gene and measuring Aβ and Tau, can be conducted if possible.

## Comprehensive AD Management

**Fig. 2:**
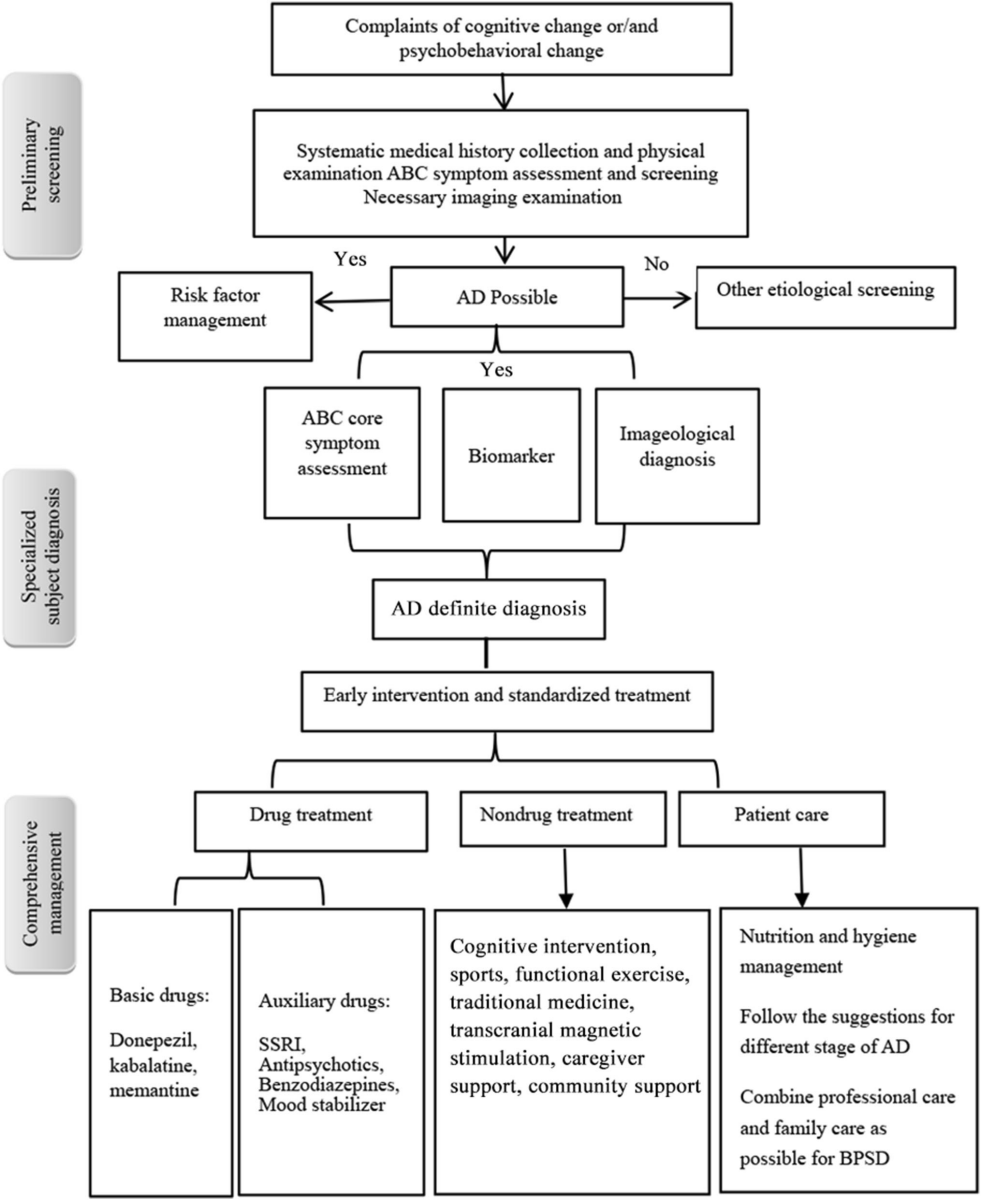
Flow chart of comprehensive ABC management for AD patients. ABC, daily living activities, behavioral and psychological symptoms, and cognitive function; AD, Alzheimer’s disease; SSRI, selective serotonin reuptake inhibitor. BPSD, behavioral and psychological symptoms of dementia.

**Fig. 3:**
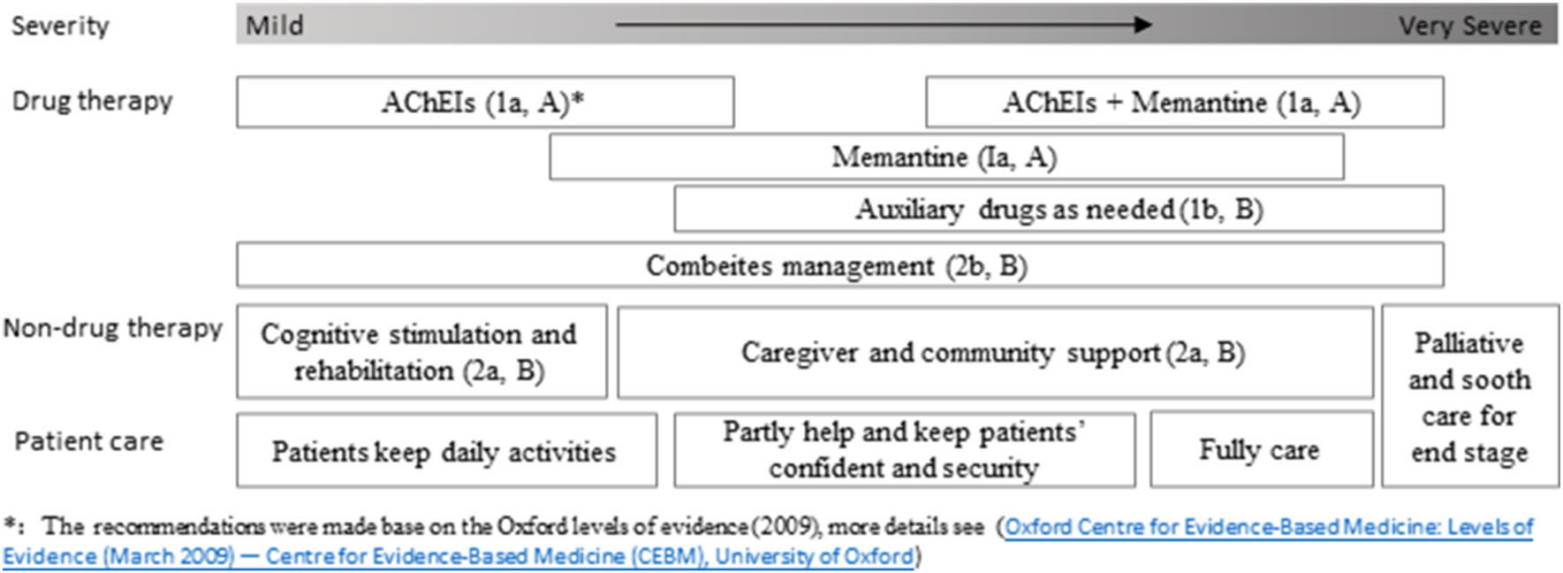
Recommendations for comprehensive management at the different stages of Alzheimer’s disease.

Hundreds of clinical studies on drugs targeting the etiologies and pathological changes in AD have failed in recent years. Therefore, the current clinical management of AD still focuses on managing the symptoms, delaying progression, improving the quality ,of life of patients, and reducing the burden on caregivers [45]. After three rounds of focus group discussions, we finished the ABC comprehensive management process instructions for AD patients to guide clinicians in optimizing diagnosis and treatment process based on the current situation (Fig. 2). For the different stages of AD, different implementations of comprehensive management are also recommended (Fig. 3).

### Positively Manage the Risk of AD and Comorbidity

Early intervention improves the clinical benefits for patients with AD [46–48]. Actively managing the risk factors, such as preventing and treating depression, quitting smoking, preventing hearing loss, stabilizing blood pressure, managing blood glucose, maintaining a reasonable diet, ensuring enough sleep, and encouraging regular physical exercise, have considerable significance in reducing and delaying the development of AD [5, 49]. After systematically reviewing the evidence of AD risk factor management, Yu *et al*. reported evidence-based suggestions on AD prevention [50].

For the elderly, comorbidities are common in AD patients (Table 4). [51–53] More studies have shown that some comorbidities are not only risk factors but also might be associated with the development of AD and affect the choice of anti-dementia medication [53, 54]. Appropriate instructions on comorbidity management are important for comprehensive management. Hypertension, hyperlipidemia, hyperglycemia, and hyperuricemia are the typical risks for AD. Studies have shown that AD patients with diabetes require less anti-dementia prescription than patients without diabetes. Diabetes may accelerate the course of cognitive decline and, apart from standard diabetes management, more attention should be paid to frequent checkups and anti-dementia treatments [55]. Chronic obstructive pulmonary disease (COPD) and asthma are common comorbidities in the elderly, who need long-term treatment with muscarinic antagonists. COPD is not only associated with the behavioral disturbances of AD patients, but also with the development of cognitive deficits and frontal deficits. Due to potential pharmacodynamic interaction between muscarinic antagonists and acetylcholinesterase inhibitors (AChEIs), AD patients with asthma/COPD might benefit more from memantine than AChEIs [53, 56]. Osteoarthritis or other chronic non-cancer pain is associated with an elevated risk of AD-related dementia; this association is particularly pronounced in those with osteoarthritis and pain, and mood disorders may partially mediate this relationship [57, 58]. Although studies have emphasized the importance of pain control for AD patients, pain management is still a dilemma because of the side-effects of opioids. The elderly with moderate-to-severe pain might benefit more from opioids than nonsteroidal anti-inflammatory drugs to achieve adequate effects, especially when patients have gastrointestinal or cardiovascular disease. The risks and benefits of investigations need to be re-evaluated for AD patients. Epilepsy management for AD patients is also a tough medical task. A 10-year nationwide cohort study showed that patients with AD have a higher risk for epilepsy than those without AD (hazard ratio =2.773, 95% confidence interval 2.515-3.057) [59]. AD patients with epilepsy need anti-epileptic and anti-dementia therapy with the same priority. Although memantine should be used cautiously in patients with epilepsy, studies with a limited sample size reported that memantine has positive effects on improving the cognitive function in epileptic patients [60–62]. The benefits and risks of memantine for AD patients with epilepsy need to be re-evaluated for the importance of N-methyl-D-aspartic acid receptor (NMDAR) over-activation during seizures.

**Table 4:** Prevalence of comorbidities in patients with Alzheimer’s disease.

Comorbidity	Prevalence
Hypertension	43.5%–55.1%
Diabetes mellitus	16.5%–25.7%
Hyperlipidemia	20.2%–40.6%
Cardiovascular disease	22.7%–60.56%
Chronic airway disease	10.2%–18.7%
Liver cirrhosis	0.50%
Chronic renal failure	0.80%–3%
Cancer	1.3%–7.8%
Gout/Hyperuricemia	5.7%–8.4%
Osteoarthritis/Pain	10.8%–38.2%
Osteoporosis	14.10%
Cerebrovascular disease	22.80%
Epilepsy	2.05%–11.7%
Parkinson’s disease	6.80%
Depression	32.3%–47.8%

### Comprehensively Manage ABC Symptoms

The ABC symptoms of patients with AD interact with each other. According to the principles of comprehensive treatment, the ABC symptoms should be taken care of comprehensively to improve the cognitive impairments, manage the psychiatric and behavioral abnormalities, ameliorate the ADL, and thus achieve greater clinical benefits for patients with AD [63]. The poor control of any ABC symptoms aggravates or influences the others.

### Apply Drug and Non-drug Therapies in Combination

Appropriate drug therapy should be selected according to the current evidence and/or expert consensus. The administration of all the anti-dementia drugs must be started from a low dose and then gradually titrated to the recommended effective or maintenance dose. Combined therapy should also be considered. Anti-psychotics are usually used at low doses and for a short time. During the treatment of AD, non-drug therapies that have certain effects in the management of ABC symptoms, are effective complements for drug therapies. Studies have shown that some non-drug therapies even delay the progression of AD [64–66].

### Stratified Patient Care of AD

For the different stages of AD, apart from comprehensively managing the ABC symptoms, suggestions for stratified care should be given to caregivers. Nutrition and hygiene require attention for all AD patients. Mild AD patients should have regular lives and participate in the social activities they enjoy to help them to maintain independent living activities at a high level for as long as possible. For moderate AD patients, caregivers should cultivate patients’ confidence and security. For severe AD patients, caregivers should particularly focus on avoiding complications such as aspiration pneumonia, pressure sores, and deep vein thrombosis. Palliative and soothing care could be considered for end-stage AD patients [45]. BPSD care is always a tough task for caregivers. Professional care training should be provided for caregivers. Professional care and family care should be combined as much as possible for patients with BPSD.

### Drug Therapy for ABC Symptom Management

Improving cognitive impairment: (a) AChEIs: The commonly used AChEIs include donepezil, rivastigmine, galantamine, and huperzine. Evidence has shown dose–effects responses in improving the cognitive impairments of AD patients but the risk of adverse responses also increases with the dose of AChEIs. Therefore, the balance between the treatment efficacy and adverse responses must be considered in clinical practice [67]. (b) NMDAR antagonists: Memantine is the major drug currently used for AD treatment. A systemic review in 2019 showed that using memantine alone effectively improves the cognitive impairments. In addition, memantine is well tolerated by patients with AD, and the incidence of adverse responses is comparable with the placebo [68].

BPSD treatment: The treatment of BPSD in patients with AD should be based on anti-dementia drugs, and anti-psychotics should also be used if necessary. Both AChEIs and memantine improve BPSD. Donepezil improves the anxiety, depression, and apathy, [69] while memantine has significant efficacy in improving the delusions, agitation, offensive behaviors, and severe stereotyped behaviors [70]. Memantine also has certain effects in preventing BPSD [71]. In addition to the application of anti-dementia drugs, anti-psychotics are only considered when non-drug therapy fails, BPSD is severe, patients do not cooperate with carers, or safety issues appear [72]. Anti-psychotics should be used singly for a short time, starting from a low dose and increasing as slowly as possible. The commonly used anti-depressive drugs mainly include citalopram, escitalopram oxalate, sertraline, and mirtazapine [73]. The commonly used atypical neuroleptics mainly include olanzapine, risperidone, quetiapine, and aripiprazole. Benzodiazepine drugs have certain anti-anxiety effects on patients with AD but more adverse responses and fewer clinical benefits than anti-psychotics. Thus, benzodiazepines should be used with caution in clinical practice [74]. Evidence regarding the effects of mood stabilizers, including valproates and lithium, on improving BPSD and cognitive impairments is still lacking [75, 76].

Preventing ADL reduction: ADL assessment is an essential factor for discriminating the severity of AD. [29] Memantine can effectively improve the BADL and IADL [77]. The effects of AChEIs on the improvement in ADL vary. For instance, donepezil and rivastigmine improve the ADL of patients in 12 weeks, while galantamine requires a longer time to exert its effects [78].

Recommendations: (1) Both AChEIs and memantine can improve the ABC symptoms in patients with AD, while combined application of the two types of drug may achieve better clinical benefits in patients with moderate or severe AD; and (2) if BPSD cannot be managed by non-drug therapy in patients receiving anti-dementia drugs, short-term application of low-dose anti-psychotics should be included.

### Non-drug Therapy for ABC Symptom Management

Non-drug therapy mainly includes cognitive intervention, BPSD management, training in ADL, physical therapy, and movement therapy, which can be used as effective complements to drug therapy and improve the ABC symptoms. Due to the high operability, as well as high acceptability by patients and families, non-drug therapy is increasingly widely applied in clinical practice.

Cognitive intervention: Cognitive intervention mainly includes cognitive training, stimulation, and rehabilitation; it combines psychological theories, methods, and gamification thinking, which can restore the absent conditioned reflex in patients with AD in the early stage. It also comprises support for caregivers [63]. Cognitive stimulation refers to a comprehensive intervention that applies stimuli for thinking, attention, and memory in community environments to improve the cognition and social functions of patients. Cognitive stimulation continuously improves global cognitive functions and the quality of life in patients with mild and moderate AD [79]. Cognitive rehabilitation uses training, including that of memory, aiming to compensate for cognitive impairments to identify and address the requirements of individuals. Cognitive rehabilitation improves the ADL of patients and reduces the burden on caregivers [80, 81].

Non-drug therapy for BPSD: The non-drug therapy for BPSD mainly includes three types: for patients, caregivers, and the environment. (a) Treatments for patients include reminiscence therapy (discussing previous experiences), validation therapy (solving previous conflicts), simulated presence therapy (using recorded sounds of family members), aromatherapy (using aromatic plant oils), music therapy, and heliotherapy. [82] (b) Treatments for caregivers reduce the BPSD of patients with AD and alleviate the burden on caregivers [83]. Individualized non-drug therapy strategies are developed *via* training and supporting caregivers while taking the interests, cognition, and physical strength of patients into consideration [84]. (c) Treatment for the environment involves eliminating factors that induce BPSD, such as preventing hyperstimulation (such as crowded and noisy environments and irritating colors) or hypostimulation (such as lacking interaction) of patients, as well as removing potential risks (such as potentially dangerous materials).

Training in ADL: ADL training delays function loss, improves the quality of life of patients, and decreases the burden on caregivers [85, 86]. During ADL training, individualized strategies should be developed considering the following factors: (a) the existing skills and interests of the patients must be preserved as much as possible; (b) the instructions for training must be concise and clear; (c) the environment and equipment must be based on the detailed conditions of patients; and (d) the caregivers are trained and participate in the training of patients [87, 88].

Movement therapy: Both aerobic exercise and resistance training are associated with decreasing the risk of cognitive impairment [89]. Movement therapy increases neuroplasticity, improves ABC symptoms, and delays disease progression. Various methods are available, and those recommended for patients with early AD mainly include jogging, Tai Chi, and gymnastics.

Physical therapy: Physical therapy includes repetitive transcranial magnetic stimulation, transcranial direct-current stimulation, photobiomodulation, and electric shock therapy. High-frequency repetitive transcranial magnetic stimulation can help improve cognitive function and address psychological and behavioral disorders, including apathy, depression, and agitation.

Other treatments: Multimodal lifestyle intervention improves the symptoms and prognoses of patients with AD [89]. The application of advanced technologies, including artificial intelligence, game tools, virtual reality, and telemedicine, play increasingly important roles in improving the quality of life of patients, as well as reducing the burdens on the families and society.

Support for caregivers: Caregivers of patients with AD are under more pressure than those with other diseases, with a heavier burden and a substantially higher tendency for depression [90, 91]. Therefore, intensifying the management and support for caregivers is necessary. Further AD knowledge should be conveyed to caregivers, effective coping strategies should be developed, and a corresponding medical insurance system and social support networks should be established. These can help alleviate the burden on caregivers, increase the quality of care for patients with AD, and improve prognoses [92, 93].

Recommendations: (1) in addition to the application of anti-dementia drugs, non-drug therapy should be given priority to manage BPSD; and (2) stratified support for caregivers is important for comprehensive management, and deserves attention.
