# Correction to: Comprehensive Management of Daily Living Activities, behavioral and Psychological Symptoms, and Cognitive Function in Patients with Alzheimer’s Disease: A Chinese Consensus on the Comprehensive Management of Alzheimer’s Disease

**DOI:** 10.1007/s12264-021-00743-3

**Published:** 2021-06-29

**Authors:** Jianjun Jia, Jun Xu, Jun Liu, Yongjun Wang, Yanjiang Wang, Yunpeng Cao, Qihao Guo, Qiuming Qu, Cuibai Wei, Wenshi Wei, Junjian Zhang, Enyan Yu

**Affiliations:** 1 Department of Neurology, The Second Medical Center, People’s Liberation Army General Hospital, Beijing, 100853 China; 2 Department of Neurology, Beijing Tiantan Hospital, Capital Medical University, Beijing, 100050 China; 3 Department of Neurology, Sun Yat-sen Memorial Hospital, Sun Yat-sen University, Guangzhou, 510120 China; 4 Cognitive Impairment Department, Shenzhen Kangning Hospital, Shenzhen, 518118 China; 5 Department of Neurology, Daping Hospital, Army Medical University, Chongqing, 400042 China; 6 Department of Neurology, The First Hospital of China Medical University, Shenyang, 210112 China; 7 Department of Gerontology, Shanghai Jiaotong University Affiliated Sixth People’s Hospital, Shanghai, 200233 China; 8 Department of Neurology, The First Affiliated Hospital of Xi’an Jiaotong University, Xi’an , 710061 China; 9 Department of Neurology, Xuanwu Hospital Capital Medical University, Beijing, 100053 China; 10 Department of Neurology, Huadong Hospital Affiliated to Fudan University, Shanghai, 200040 China; 11 Department of Neurology, Zhongnan Hospital of Wuhan University, Wuhan, 430071 China; 12 Department of Psychology, Chinese Academy of Sciences Cancer Hospital of the University of the Chinese Academy of Sciences, Hangzhou, 310022 China

## Correction to: Neuroscience Bulletin (2021) 10.1007/s12264-021-00701-z

The article ‘Comprehensive Management of Daily Living Activities, behavioral and Psychological Symptoms, and Cognitive Function in Patients with Alzheimer’s Disease: A Chinese Consensus on the Comprehensive Management of Alzheimer’s Disease’, written by Jun Xu, Jun Liu, Yongjun Wang, Yanjiang Wang, Yunpeng Cao, Qihao Guo, Qiuming Qu, Cuibai Wei, Wenshi Wei, Junjian Zhang & Enyan Yu, was originally published electronically on the publisher’s internet portal on 29th May 2021 without open access. With the author(s)’ decision to opt for Open Choice the copyright of the article changed on 11th June 2021 to © The Author(s) 2021 and the article is forthwith distributed under a Creative Commons Attribution 4.0 International License, which permits use, sharing, adaptation, distribution and reproduction in any medium or format, as long as you give appropriate credit to the original author(s) and the source, provide a link to the Creative Commons licence, and indicate if changes were made. The original article has been corrected.

